# Worry and Positive Episodes in the Daily Lives of Individuals With Generalized Anxiety Disorder: An Ecological Momentary Assessment Study

**DOI:** 10.3389/fpsyg.2021.722881

**Published:** 2021-10-05

**Authors:** Andreea Vîslă, Richard Zinbarg, Peter Hilpert, Mathias Allemand, Christoph Flückiger

**Affiliations:** ^1^Department of Psychology, University of Zürich, Zürich, Switzerland; ^2^Department of Psychology, Northwestern University, Evanston, IL, United States; ^3^Department of Psychology, University of Lausanne, Lausanne, Switzerland

**Keywords:** worry episodes, positive episodes, anxiety, uncontrollability, event-based ecological momentary assessment, generalized anxiety disorder

## Abstract

Worry is a central feature of generalized anxiety disorder (GAD). Although worry is related to anxiety and maintained by beliefs that worry is uncontrollable, there is scarce research on how individuals with GAD react to worry episodes in their daily life and how their positive experiences might impact reactions to worry episodes. The current study examined the level and variability of anxiety and controllability during high worry periods and positive experiences in GAD. Moreover, it investigated the influence of worry and positive experiences on later anxiety and perceived controllability within-persons. Finally, it examined change in anxiety level from previous to current episodes depending on previous episodes type. In the current study, 49 individuals with GAD (514 observations) registered their worry and positive episodes (i.e., episodes in which they had positive experiences) and reported on several variables during these episodes (i.e., anxiety and controllability of episodes and episode duration) using smartphone-based ecological momentary assessment for 7days. Results show anxiety and controllability differed by episode type (higher anxiety, lower controllability in worry episodes, and the opposite in positive episodes), and notable within-person variability in anxiety and controllability in both episode types. The time-lagged multilevel models showed episode type did not predict later anxiety during either episode type, although previous anxiety predicted current anxiety in worry episodes (but not positive episodes). Moreover, worry episodes did predict later controllability in worry episodes (but not positive episodes) and previous controllability predicted current controllability in both episode types. Furthermore, we obtained the increase in anxiety from *t*_0−1_ to *t*_0_ in a current worry episode to be significantly smaller when preceded by a worry (vs. positive) episode. Likewise, the reduction in anxiety from *t*_0−1_ to *t*_0_ in a current positive episode was significantly larger when preceded by a worry (vs. positive) episode. The novel findings in the current study that perceptions of controllability and anxiety vary within individuals with GAD, that greater controllability is experienced in positive episodes than worry episodes, and that worry may confer a sense of controllability at a later time could be seen as important contributions to the GAD literature.

## Introduction

Traditionally, clinical assessment has focused on symptoms, deficits, and disorders. However, there have been some attempts to incorporate individuals’ positive factors into the assessment and treatment of mental disorders (e.g., Gelso and Woodhouse, 2003; Seligman and Peterson, 2003; Duckworth et al., 2005; Rashid and Ostermann, 2009; Scheel et al., 2013). The current study explores how individuals with generalized anxiety disorder (GAD) react to worry and positive episodes (i.e., episodes in which they had positive experiences) in their daily life and how these episodes impact each other within-person.

Excessive worry is one of the main GAD diagnostic criteria (DSM-5; American Psychiatric Association, 2013). Worry is a type of repetitive negative thinking (Ehring and Watkins, 2008; Wahl et al., 2019), defined as a relatively uncontrollable chain of primarily verbal-linguistic thoughts about uncertain events with the potential for future negative outcome (Borkovec, 1994). In a recent meta-analysis (*k*=138) of both cross-sectional and longitudinal studies, worry has been found to be strongly associated with negative affect (e.g., anxiety and general distress; *r*=0.55, *p*<0.001) and moderately associated with low positive affect and wellbeing (*r*=−0.23, *p*<0.001) in various populations, including individuals with GAD (Vîslă et al., in review). Moreover, few experimental studies found worry induction to increase self-reported anxiety and depression in individuals with GAD (e.g., Llera and Newman, 2010, 2014). Using intensive-longitudinal designs, weekly worry predicted weekly negative emotions in healthy adolescents (Dickson et al., 2012) and a subclinical sample of students (Crouch et al., 2017). In a more recent study, Newman et al. (2019) investigated worry in GAD individuals using a time-based ecological momentary assessment (EMA) with 10 prompts per day for 8days and found higher worry duration and negative thought valence to predict feeling concurrently (but not sustained 1h later) keyed up (Newman et al., 2019). Whereas worry is a central process in GAD that maintains anxiety and psychological distress, there is a claim for future research to better understand the factors that contribute to maintaining or diminishing the anxiety across worry episodes (e.g., Newman and Przeworski, 2018; Newman et al., 2019).

The Contrast Avoidance model of GAD (CAM; Newman and Llera, 2011; Llera and Newman, 2014) suggests that worry serves the purpose of limiting reactivity to abrupt changes in emotional states, such as switching from a neutral or positive emotion to a negative emotion. Specifically, CAM posits that the main symptom of GAD, i.e., worry, has the role to avoid negative contrasts (i.e., shifts from positive to negative emotions) and to increase the probability of positive contrasts (i.e., shifts from negative to positive emotions) in order to maintain constant negative affect levels. Therefore, worry is used to increase and maintain anxiety, particularly after a positive state (generated by a positive experience or event) that is vulnerable to shifts. For those with GAD, the positive emotions or low anxiety in a positive episode are experienced as vulnerable to later emotional shifts. Worry reduces this vulnerability by creating anxious feelings, lessening, or stopping the positive emotional carry-over from prior positive events.

Although worry episodes are present to some degree in all individuals, the uncontrollability of these episodes seems to distinguish worry in individuals with GAD from worry in other anxiety disorders (Hoyer et al., 2001; Wells and Carter, 2001), subsyndromal worry (Wetherell et al., 2003; Ruscio and Borkovec, 2004; Hirsch et al., 2013), and normal worry (Abel and Borkovec, 1995; Hoyer et al., 2001; Wells and Carter, 2001; Wetherell et al., 2003). Moreover, some studies found inability to control worry to be the only distinct phenomenon among persons with GAD compared to others (Craske et al., 1989; Ruscio and Borkovec, 2004). Additionally, worry uncontrollability has been shown to contribute to the validity of GAD diagnosis, even after controlling for worry excessiveness (Hallion and Ruscio, 2013). In several longitudinal studies, uncontrollability beliefs predicted daily worry, above and beyond intolerance of uncertainty and trait worry (Thielsch et al., 2015). Furthermore, changes in uncontrollability beliefs across treatment predicted concurrent changes in repetitive negative thinking (McEvoy et al., 2015). In a randomized-controlled trial comparing two ecological momentary interventions for GAD, higher uncontrollability beliefs predicted worse treatment outcome (LaFreniere and Newman, 2019). Taken together, although the belief in the need for control is a central feature of anxiety disorders in general (Rapee et al., 1996; Mineka and Zinbarg, 2006), the belief that worry episodes are uncontrollable is a key feature of individuals with GAD, with some indications that decreasing this belief might reduce worry in individuals with GAD.

Although individuals with GAD might find it hard to control their worries, they might, at the same time, hold the belief that worry is helpful (Dugas et al., 1998) in avoiding negative emotional contrast (Newman and Llera, 2011). This might give those with GAD a sense of high controllability over worry episodes and their associated emotional reactions. Positive experiences (accompanied by positive emotions and/or low anxiety), on the other side, could offer individuals with GAD the feeling that they have less control, since there is a high probability of negative contrasts. Indeed, for individuals with GAD, the positive emotions in a positive episode (or low anxiety) are experienced as vulnerability to later emotional shifts (Newman and Llera, 2011).

Although clinical assessment is mainly focused on identifying symptoms, some research has suggested incorporating an assessment on individuals’ strength, i.e., what strengths does the individual bring to deal effectively with his or her problems in daily life, e.g., positive experiences, thinking (e.g., hope and positive expectations), and emotions (Fredrickson, 2001; Seligman and Peterson, 2003; Duckworth et al., 2005; Rashid and Ostermann, 2009; Scheel et al., 2013). From a strength-based assessment perspective, anxiety disorders and implicitly GAD might not only represent worrying, feeling restless, and lacking focus, but also it could be a lack of purposeful goals and actions that consume individuals’ resources (Seligman and Peterson, 2003; Cheavens et al., 2006; Grawe, 2006; Rashid and Ostermann, 2009; Flückiger et al., 2014). The tendency of individuals with GAD to prevent negative outcomes could, however, be seen as a very purposeful goal (Newman and Llera, 2011). Nevertheless, such prevention goals are usually different from approach and promotion goals, e.g., how to avoid danger vs. how to engage in rewarding activities and fulfill ideas and hopes (Higgins, 1998; Strauman et al., 2015). Moreover, the broaden-and-build theory (Fredrickson, 2001) posits that positive emotions (that accompany positive experiences or events) may foster the activation of personal strengths and resources in individuals that might benefit their overall mood (e.g., increase positive emotions and/or decrease anxiety).

In spite of some lasting theoretical considerations and some attempts to integrate patient strengths into the assessment and treatment of mental disorders (e.g., Fredrickson, 2001; Gelso and Woodhouse, 2003; Seligman and Peterson, 2003; Cheavens et al., 2006; Rashid and Ostermann, 2009; Scheel et al., 2013), there is limited empirical research exploring the role positive experiences might have in diminishing symptoms. Experimental research generally found depressed individuals to be less reactive to positive stimuli than nondepressed individuals (e.g., Bylsma et al., 2008; Dichter, 2010). However, in EMA studies, depressed individuals reported larger increases in positive affect (Peeters et al., 2003; Khazanov et al., 2019) and larger declines in negative affect (Peeters et al., 2003; Bylsma et al., 2011; Thompson et al., 2012; Khazanov et al., 2019) than nondepressed individuals following positive events in their daily lives. In individuals with GAD, just one study examined the influence of positive experiences using EMA on affect and did not find such a strong “brightening effect” in those with GAD as in those with major depression (Khazanov et al., 2019). In this study, major depression severity was a stronger predictor of “brightening” than GAD severity (Khazanov et al., 2019). Therefore, given the tendency of individuals with GAD to worry in order to avoid negative contrasts in emotions and to increase the probability of positive contrasts, investigating how they react to positive experiences in the moment and the influence these positive experiences have on later anxiety and controllability might have important theoretical and clinical implications.

Studies have revealed significant discrepancies between real-time assessments and retrospective self-reports of mood, symptoms, and behaviors across a range of clinical problems (Solhan et al., 2009; Moore et al., 2016). In a recent study using both retrospective self-reports and time-based EMA, Mathersul and Ruscio (2020) showed that GAD severity was associated with negative memory biases, i.e., individuals with GAD remembered past episodes as more negative than they actually reported them in the EMA. Therefore, EMA might be a complementary assessment approach for investigating intraindividual processes over time within-person in general (Bolger et al., 2003; Hamaker, 2012; Fisher and Bosley, 2020) and in GAD more specifically (Newman and Przeworski, 2018). As presented above, there is some research in GAD using intensive-longitudinal designs with time-based assessment of worry and negative affect (Newman et al., 2019) and emotional reactions to positive experiences (Khazanov et al., 2019). While time-based designs are usually used to capture some clinical phenomena that vary continuously, for example, mood, interest in particular events or episodes, e.g., worry episodes, are usually captured using event-based assessment, in which assessments are triggered by the occurrence of a predefined event of interest for the investigator (Shiffman et al., 2008).

### The Current Study

The present study used event-based (participant-initiated) EMA for 7days to examine worry and positive episodes experienced by individuals with GAD in their daily life, before starting therapy. First, we aimed to investigate whether individuals with GAD experience their worry episodes with more anxiety and less controllability than their positive episodes in the moment.

Second, we aimed to investigate at the within-person level whether the type and level of the previous episode (*t*_0−1_) predict anxiety and controllability level in the current episode (*t*_0_). Based on the assumptions of the CAM (Newman and Llera, 2011; worry is stopping or lessening the positive emotional carry-over from prior positive events), we expected previous episode type will not predict later anxiety during either current episode type. The investigation of previous episode type predicting current controllability was exploratory (since no worry model makes an explicit assumption about perceived controllability). Moreover, we expect previous episode severity will predict severity in the next episode.

Third, we investigated whether the change in anxiety level from a previous episode to a current episode (*t*_0−1_->*t*_0_) is predicted by the type of the previous episode. Therefore, we investigated as: (a) whether the increase in anxiety from *t*_0−1_ to *t*_0_ in a *current worry episode* is significantly smaller when preceded by a worry (vs. positive) episode and (b) whether the reduction in anxiety from *t*_0−1_ to *t*_0_ in a *current positive episode* is significantly larger when preceded by a worry (vs. positive) episode.

## Materials and Methods

### Participants

The participants included in this study were individuals with GAD that were selected to take part later in a randomized-controlled trial conducted at the University of Zurich. The GAD diagnosis and its core symptomatology were identified by trained interviewers according to the German structured interview section for DSM-5 (DIPS, Margraf et al., 2017) by trained and supervised interns with at least a Bachelor’s degree in Clinical Psychology. Patients were only included in the study if all three evaluations (self-evaluation, phone screening, and structured interview) agreed on a GAD diagnosis. Interrater agreement of the GAD diagnoses of structured interview was 95%; in the few cases of disagreement, the participants were excluded from the study. The primary aim of the trial was to investigate the enduring efficacy of cognitive-behavioral therapy for GAD (Flückiger et al., 2021 Clinical trial registration: ClinicalTrials.gov #NCT03079336). Inclusion criteria were as: (1) meeting *Diagnostic and Statistical Manual of Mental Disorders* (DSM-5; American Psychiatric Association, 2013) criteria for GAD; (2) being at least 18years old; and (3) giving informed consent. Exclusion criteria were as: (1) a score of 2 or higher on the suicide item of the Beck Depression Inventory and/or active suicidal plans during the diagnostic screening interview, (2) current medication for psychotic or bipolar disorder, or (3) current treatment from a professional psychotherapist. Prescribed medications for anxiety or depressive disorders did not lead to exclusion from the study if the dosage has remained constant for at least 1month. The presence of comorbidities did not result in exclusion from the study if GAD was the primary diagnosis. For more details about the selection procedure, see Flückiger et al. (2021).

Fifty-five individuals with GAD agreed to take part in the smartphone-based EMA. The rationale given to the participants for engaging in the EMA was that the data will be used, besides its research propose, to build a report that their therapists will use to customize the treatment to their individual needs. Of the 55 participants who began the study, two participants’ data were lost due to technical problems with the smartphones, two individuals did not return the smartphone, and two participants did not comply with EMA due to time conflicts (e.g., holidays). The final sample included 49 participants (514 observations) whose data were used for the present study analyses. Demographics and clinical variables of the included participants are shown in Table 1. The sample included in the current analyses did not differ from the six dropouts in any of the characteristics included in Table 1 (*p*>0.19).

**Table 1:** Demographic and clinical variables of the included participants.

	*M*	*SD*	*n*	%
Age	29.18	8.58	49	
Gender				
Male			14	29
Female			35	71
Years with generalized anxiety disorder^1^	11.05	10.47	42	
Comorbidity				
No			30	61
Yes			19	39
Previous diagnosis/diagnoses				
No			37	76
Yes			12	24
Marital status				
Married			10	20
Not currently married			39	80
Educational status				
College			4	8
University Bachelor			15	31
University Master			11	22
PhD			18	37
Unknown			1	2
Employment status				
Full-time			15	31
Part-time			29	59
Unemployment^2^			5	10
Foreign background			18	37

1 This information was not available for seven participants.

2 In this category, students without a part-time job were also included.

*M*=mean; *SD*=standard deviation; and *n*=number of participants included in the specific analyses; Unknown is the number of missing data for the specific categories included.

### Assessments and Procedure

EMA was introduced in the randomized clinical trial after the intake phase, when a GAD diagnosis was established, and just before the therapy started. EMA was implemented using a smartphone application installed on Motorola smartphones (eXperience Sampling for Android by MovisensXS).^1^ Data using EMA were collected for 7days using event-based (participant-initiated) assessment, meaning that participants were instructed to report each experienced episode. Each participant was called by a team member to clarify any questions regarding the use of the smartphone and EMA app. Participants were also given the possibility to call the contact person for any question or possible problems they might encounter while using the EMA app. We observed privacy rights and obtained informed consent from study participants. The study was approved by the Ethical Committee of University of Zurich (BASEC 2016-00773).

During the initial phone call and in written instructions, participants were instructed to report their worry and positive episodes right after they took place. For every registered episode, participants were asked to report the type of the episode they just experienced, i.e., worry or positive episode. Episodes were defined to the participants as follows:

A *worry episode* is an episode in which you experienced a significant amount of worries. Worries are repetitive and disturbing thoughts about future events that are associated with negative emotions when they occurred. These worry episodes can be related to one or more domains in your life (for example, relationships and health) and you can experience them both when you are alone or with other people (Mclaughlin et al., 2007).

A *positive episode* is an episode in which you had positive life experiences. Positive experiences are experiences that have a positive impact on you, for example, are associated with positive thoughts and positive emotions. These positive episodes can be experienced alone or with others (Duckworth et al., 2005).

For every reported worry and positive episode, the participants filled in information about the duration of the episode (in minutes), the anxiety experienced in the episode and the perceived controllability over the experienced episode. The anxiety level was assessed using the question: “To what extent are you experiencing anxiety?”, with the answers rated on a visual analog scale ranging from “no anxiety” (=0) to “extreme anxiety” (=100). Perceived controllability regarding the current experienced episode was assessed using the control scale of the Self-Assessment Manikin (SAM; Bradley and Lang, 1994). The SAM is a picture-oriented questionnaire developed to measure the pleasure, arousal, and control associated with an individual’s reaction to an episode and has been successfully used in other studies to assess these dimensions (Backs et al., 2005; Hur et al., 2019). The control scale of the SAM contains an item evaluating the extent to which the individuals think they are in control of the episode they are currently experiencing and is rated on a 9-Likert scale, ranging from 1 (“not at all in control”) to 9 (“totally in control”). We used single-item measures to minimize the participant burden (lessen their required effort) in case many episodes occurred (single items are quicker). When we will know more about the baseline rate of these episodes, measurements with more items can be implemented. Additionally, the date and time of the reported episodes were automatically registered by the EMA app.

### Statistical Analyses

The current study had three aims. First, we examined how individuals with GAD perceive their worry vs. their positive episodes in the moment in terms of anxiety and perceived controllability. Second, we investigated at the within-person level whether the type and level of the previous episode (*t*_0−1_) predict anxiety and perceived controllability in the current episode (*t*_0_). Third, we investigated whether the change in anxiety level from a previous episode to a current episode (*t*_0−1_->*t*_0_) is predicted by the type of the previous episode. As these data are nested (repeated measures nested in a person; Bolger and Laurenceau, 2013), a multilevel modeling approach was used to examine the hypotheses.

Before conducting the analyses, we determined the level of nonindependence in our dependent variables (i.e., anxiety and controllability in the current episode) by estimating a null model and calculating the intraclass correlation coefficient (ICC) at individual level (i.e., within- and between-person variances) for worry episodes and positive episodes. To answer the first research question, we computed two random-intercept multilevel models. In these models, anxiety and controllability were predicted by the episode type (i.e., positive episode=0; worry episode=1; and anxiety and controllability scores reported in each episode were nested in individuals).

To answer our second research question (Models 1–4), four similar time-lagged random-intercept multilevel models with restricted-maximum likelihood were fitted. Model 1 examined how *anxiety in a current worry episode* at *t*_0_ is predicted by the type of the previous episode (i.e., positive episode=0; worry episode=1) and anxiety in the previous episode. The equation for this first model is indicated here:


$${\text{anxietyWE}}_{t0}=\gamma_{00}+\gamma_{01}\left({\text{episode}}_{t0-1}\right)+\gamma_{02}\left({\text{anxiety}}_{t0-1}\right)+u_{0i}$$ (1)

**Figure 1:**
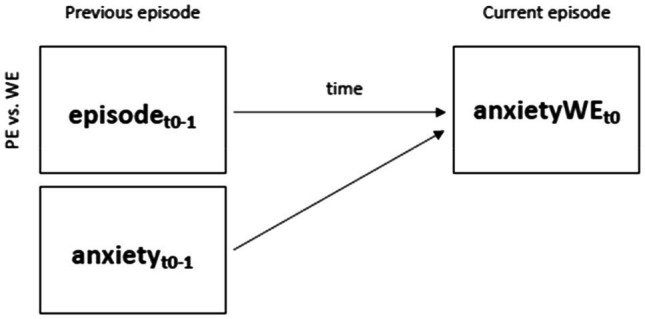
Investigating the impact of previous episode type (i.e., positive episode vs. worry episode; *t*_0−1_) on anxiety in a current worry episode (*t*_0_) using time-lagged models (anxiety at *t*_0−1_ and time between previous and current episode was added as covariates in the model; H2a). PE=positive episode; NE=negative episode; and time=time between previous (*t*_0−1_) and current episode (*t*_0_).

In this model, anxietyWE_*t*0_ indexes the anxiety in a worry episode at the current event (*t*_0_) as outcome variable; *γ*_00_ indicates the intercept, *γ*_01_ captures the effect of previous episode type (i.e., positive episode=0; worry episode=1; *t*_0−1_), *γ*_02_ represents the effect of the anxiety at the previous episode (*t*_0−1_), and *u*_0*i*_ captures the random intercepts.^2^ The following three models testing Models 2–4 are structurally identical; they only differ in the dependent variable that was predicted (i.e., anxiety or controllability at *t*_0_) and the current episode in which the dependent variable was reported (i.e., worry or positive episode at *t*_0_). Model 2 predicts the *controllability in a current worry episode*, model 3 – the *anxiety in a current* positive *episode*, and model 4 – the *controllability in a current* positive *episode* (for a graphical illustration of Model 1, see Figure 1).

To answer our third research question, whether change in anxiety from previous episode (*t*_0−1_) to current episode (*t*_0_) is predicted by previous episode type, we computed two models in which change was predicted by previous episode type (i.e., positive episode=0; worry episode=1) in a current worry episode (Model 5) and a current positive episode (Model 6). Based on an *a priori* power analyses for a traditional MANOVA design with repeated measurements, it was determined that a sample size of at least 34 participants was required to observe a medium effect size of 0.5 with an alpha level of 0.05 (two tailed) and power of 0.8 (Erdfelder et al., 1996).

The R “multilevel” package (Bliese, 2016) was used for computing the ICC coefficients, the “dplyr” package (Wickham et al., 2019) for building the lag variable, and the “lme4” package (Bates et al., 2015) for running the multilevel models.

## Results

### Preliminary Analyses

Our data set contained 514 reported episodes (331 worry episodes and 183 positive episodes) nested in 49 individuals (*M*=10.49, *SD*=9.11, range=1–36)^3^, with each individual being assessed over 7 days. On average, participants documented their episodes during 4.2days^4^ (*SD*=2.19; range=1–7). Compliance with the assessed items was 99%; participants self-initiated the events, meaning that the compliance reported here describes the compliance to the prompted items during an event self-initiated by the participant. The average duration of the worry episodes per individual was *M*=105.3min^5^ (*SD*=563.5, range=1–1800) and the average duration of the positive episodes per individual was *M*=80.2min (*SD*=93.7, range=1–540). In 43% of the cases, a worry episode was preceded by a worry episode and a positive episode preceded worry episode in 22% of the cases. Moreover, a positive episode was preceded by a positive episode in 13% of the cases and in 22% of the cases, a worry episode preceded a positive episode.

For *worry episodes*, the ICC showed that individuals explained ICC=0.31 of the variance in anxiety and ICC=0.34 of the variance in the controllability reported during current worry episodes. Moreover, the within-person variance in anxiety (ICC=0.69) and controllability (ICC=0.66) was significantly higher (*p*<0.001) than the between-person variance in anxiety (ICC=0.31) and controllability (ICC=0.34). For *positive episodes*, the ICC showed that individuals explained ICC=0.49 of the variance in anxiety and ICC=0.32 of the variance in the controllability reported during current positive episodes. Moreover, as for the current worry episodes, the within-person variance in anxiety (ICC=0.51) and controllability (ICC=0.68) was significantly higher (*p*<0.001) than the between-person variance in anxiety (ICC=0.49) and controllability (ICC=0.32) during current positive episodes.

### Momentary Reactions to Worry Episodes and Positive Episodes

The average anxiety reported across all individuals during their worry episodes (*M*=62.68; *SD*=20.21; range=0–100) was significantly higher than of the average anxiety reported across all individuals during their positive episodes (*M*=22.73; *SD*=17.89; range=0–69; *ß*=38.57, *SE*=1.65, *t*-value=23.45, *p*<0.001). This result indicates that participants experienced more anxiety in the worry episodes than in the positive episodes. The average of perceived controllability reported during the worry episodes (*M*=3.69; *SD*=1.44; range=1–8) was significantly lower than the average controllability reported during the positive episodes (*M*=6.56; *SD*=1.17; range=2–9; *ß*=−2.86, *SE*=0.11, *t*-value=−25.49, *p*<0.001). This result indicates that participants experienced less controllability in the worry episodes than in the positive episodes.

### Influence of Previous Episode Type and Level (*t*_0−1_) on Level of Current Episode (*t*_0_)

Results are presented in Table 2. In Model 1, we found higher previous anxiety level to predict higher current anxiety level (*p*=0.019; Cohen’s *d*=1.6). However, in this model, previous episode type (i.e., positive vs. worry episode) did not predict the *anxiety* level experienced in a *current worry episode* (*p*=0.191).

**Table 2:** Multilevel models’ results testing the influence of previous episode type and level (*t*_0−1_) on the level of the current episode (*t*_0_).

	*γ*	*SE*	*t*	*p*
**Model 1: Anxiety in WE*t*_0_^a^**				
Intercept	58.81	3.16		
Episode *t*_0−1_	−3.9	2.98	1.31	0.191
Anxiety *t*_0−1_	0.15	0.06	2.46	0.019
**Model 2: Controllability in WE*t*_0_^b^**				
Intercept	2.06	0.41		
Episode *t*_0−1_	0.45	0.21	2.09	0.037
Controllability *t*_0−1_	0.26	0.06	4.14	<0.001
**Model 3: Anxiety in PE*t*_0_^c^**				
Intercept	28.58	4.10		
Episode *t*_0−1_	−5.73	3.45	−1.66	0.099
Anxiety *t*_0−1_	0.02	0.06	0.24	0.809
**Model 4: Controllability in PE*t*_0_^d^**				
Intercept	5.40	0.53		
Episode *t*_0−1_	0.57	0.28	2.03	0.044^e^
Controllability *t*_0−1_	0.19	0.08	2.44	0.017

a Model 1: Prediction of anxiety experienced in a current worry episode (303 observations from 44 individuals).

b Model 2: Prediction of controllability experienced in a current worry episode (303 observations from 44 individuals).

c Model 3: Prediction of anxiety experienced in a current positive episode (162 observations from 39 individuals).

d Model 4: Prediction of controllability experienced in a current positive episode (162 observations from 39 individuals).

e Having the time between previous and current episode included in these models did not change the results of these models, with the exception of the model predicting controllability in current positive episodes, for which the influence of previous episode type became non-significant (*p*=0.07).

WE=worry episode; PE=positive episode; *t*_0_=current episode; *t*_0−1_=previous episode; and episode *t*_0−1_=type of the previous episode (i.e., positive episode=0; worry episode=1).

In Model 2, previous worry (vs. positive) episode predicted higher controllability level in a current worry episode (*p*=0.037; Cohen’s *d*=2.1). Moreover, higher previous controllability level predicted higher current controllability level (*p*<0.001; *d*=0.3).

In Model 3, neither the type of the previous episode (*p*=0.099) nor the previous anxiety level (*p*=0.809) predicted the *anxiety* level in the *current positive episode*.

Finally, in Model 4, previous worry (vs. positive) episode predicted higher controllability level in a current positive episode (*p*=0.044; Cohen’s *d*=2.6); of note, when the time between episodes (i.e., time between previous and current episode) was included as a predictor in this model, the influence of episode type became non-significant (*p*=0.07). However, higher previous controllability level predicted higher current controllability level in this model (*p*=0.017; Cohen’s *d*=1.8)^6^.

### Influence of Previous Episode Type (*t*_0−1_) on Change in Anxiety From Previous to Current Episode (*t*_0−1_->*t*_0_)

The type of the previous episode (*t*_0−1_) was a significant predictor of the change in anxiety from previous episode to current episode (*t*_0−1_->*t*_0_), for both current worry episode and current positive episode (see Table 3). Specifically, the increase in anxiety from *t*_0−1_ to *t*_0_ in a *current worry episode* was significantly smaller when preceded by a worry (vs. positive) episode (*p*<0.001). Moreover, the reduction in anxiety from *t*_0−1_ to *t*_0_ in a *current positive episode* was significantly larger when preceded by a worry (vs. positive) episode (*p*<0.001).

**Table 3:** Multilevel models’ results testing the influence of previous episode type (*t*_0−1_) on change in anxiety from previous to current episode (*t*_0−1_->*t*_0_).

	*γ*	*SE*	*t*	*p*
**Model 5: Anxiety from *t*_0−1_ to WE*t*_0_^a^**				
Intercept	38.68	2.30		
Episode *t*_0−1_	−36.41	2.83	−12.88	<0.001
**Model 6: Anxiety from *t*_0−1_ to PE*t*_0_^b^**				
Intercept	2.45	2.74		
Episode *t*_0−1_	−44.71	3.48	−12.84	<0.001

a Model 5: Prediction of anxiety experienced in a current worry episode (303 observations from 44 individuals).

b Model 6: Prediction of anxiety experienced in a current positive episode (162 observations from 39 individuals).

WE=worry episode; PE=positive episode; *t*_0_=current episode; *t*_0−1_=previous episode; and episode *t*_0−1_=type of the previous episode (i.e., positive episode=0; worry episode=1).

## Discussion

The present study is the first that we are aware of that examined reactions to worry and positive episodes in the daily lives of individuals with GAD using smartphone-based EMA. Specifically, we investigated how individuals with GAD react, in terms of anxiety and perceived controllability, to worry vs. positive episodes in the moment. Moreover, we examined the impact of worry and positive experiences on later anxiety and perceived controllability within-persons. Finally, we investigated change in anxiety level from previous to current episode depending on previous episode type. Results show anxiety and controllability differed by episode type (higher anxiety, lower controllability in worry episodes, and the opposite in positive episodes), and notable within-person variability in anxiety and controllability in both episode types. The time-lagged multilevel models showed episode type did not predict later anxiety during either episode type, although previous anxiety predicted current anxiety in worry episodes (but not positive episodes). However, worry episodes did predict later controllability in worry episodes (but not positive episodes) and previous controllability predicted current controllability in both episode types. Moreover, change in anxiety level from previous to current episode depended on the type of the previous episode.

We found that individuals with GAD reported more momentary anxiety and less controllability during their worry episodes than during their positive episodes, on average (although there was variability in anxiety and controllability within episode type). These observations are consistent with correlational and experimental research showing worry to be associated with and to induce significant anxiety levels (Llera and Newman, 2010, 2014; Vîslă et al., in review). In a study using EMA, Newman et al. (2019) found higher worry duration to predict feeling concurrently keyed up. Moreover, these results align with research showing low perceived controllability over worry episodes to be a characteristic of individuals with GAD (Ruscio and Borkovec, 2004; Hallion and Ruscio, 2013). Furthermore, these findings are consistent with the assumptions of the broaden-and-build theory (Fredrickson, 2001), i.e., positive experiences may foster the activation of personal strengths and resources in individuals that might benefit their overall mood (i.e., decrease anxiety and/or increase positive affect), and with research discussing the role of positive experiences in symptom reduction during psychotherapy (Seligman and Peterson, 2003; Cheavens et al., 2006; Rashid and Ostermann, 2009; Flückiger et al., 2010). What our study adds to the previous research, however, is a comparison of anxiety and controllability levels over different life contexts (when experiencing worry vs. positive episodes). Thus, our preliminary findings show that these variables are not stable (i.e., either low or high all the time, as often assumed in retrospective self-reports and trait measures), but rather that they fluctuate depending on individuals’ life contexts (Mineka and Zinbarg, 2006). Descriptively, the current study shows the presence of not only psychological symptoms in individuals with GAD, but also of positive experiences (although less frequent than the symptoms). This is still a meaningful finding, considering that the participants in this study reported on their worry and positive episodes *before* they started therapy.

Our results showing episode type (i.e., worry vs. positive episode) does not predict later anxiety during either episode type are consistent with the study of Khazanov et al. (2019) and with the CAM (Newman and Llera, 2011). The only study that examined the influence of positive experiences using EMA on affect in individuals with GAD, Khazanov et al. (2019) found no “brightening” effect of positive events in individuals with GAD; it was even more so the case when MDD symptoms were controlled for in those with GAD. For individuals with GAD, the positive emotions or low anxiety in a positive episode are experienced as vulnerable to later emotional shifts (Newman and Llera, 2011). According to the CAM, worry reduces this vulnerability by creating anxious feelings, stopping, or lessening the positive emotional carry-over from prior positive events. As our results (discussed below) suggest, it may confer a sense of controllability, which may be more valued by those with GAD than positive emotions or even low anxiety. Therefore, worry stops positive emotion and maintains high anxiety to buffer shifts; it functions to cutoff positive episode “carry-over”, which makes those with GAD feel vulnerable.

The increase in anxiety from a previous episode to a *current worry episode* was smaller when the previous episode was a worry (vs. positive) episode.

The reduction in anxiety from a previous episode to a *current positive episode* was larger when the previous episode was a worry (vs. positive) episode.

Although episode type (i.e., worry vs. positive episode) did not predict later anxiety during either episode type, it did impact, nevertheless, its controllability level. Specifically, a previous worry (vs. positive) episode predicted higher perceived controllability during a next worry episode. Although the exact reason for such results remains unknown, we offer some speculations. It is plausible that the experience of one worry episode after the other offers individuals with GAD the impression that they are in control of their worry episode (and implicitly of their emotions experienced during these episodes) compared to when they experience a shift from a positive to a worry episode. Therefore, this finding suggests one function of worry may be to confer a sense of controllability to the worrier. This is not entirely or directly captured by any of the current most supported functional models of worry, such as the CAM (Newman and Llera, 2011) or the Intolerance of Uncertainty model (Dugas et al., 1998), though indirectly suggested by them perhaps. Therefore, it could be that worrying has not only the role of keeping a constant level of negative emotions (as postulated by the CAM), but also might function as a way of keeping a high sense of controllability over worry episodes and their associated emotional reactions. One possible factor that might intervene in the maintenance of this impression of controllability is positive beliefs about worry. According to the Intolerance of Uncertainty model of GAD (Dugas et al., 1998; see, e.g., Bottesi et al., 2016 for research supporting this model), positive beliefs about worry are distorted beliefs about the usefulness of worrying that contribute to the maintenance of worry. Therefore, it could be that the impression of control over worry is influenced by the positive beliefs that worry is helpful. Of course, the presently novel findings suggesting one function of worry is to confer a sense of controllability to the worrier require additional testing before any firm conclusions with implications for theory and practice are made.

We note that although worry episodes were experience as less controllable in the moment, worry predicted later controllability in a worry episode. We believe that worry might be experienced as less controllable in the moment because it might follow a positive event or experience that could bring a negative shift in emotions. However, a worry episode after the other might give the worrier the feeling he has control over his worry. This might be the case because constant worrying (in this case, a worry episode followed by another worry episode) might have the function to increase the probability of a positive contrast. Therefore, the expected high chances of the worried to experience a positive contrast when worrying “for a while” might be one factor that explains the high controllability in a worry episode followed by another worry episode.

When looking if episode type predicted how anxiety changed until the next episode, we found increase in anxiety was smaller for a worry (vs. positive) episode followed by a worry episode. Moreover, we found reduction in anxiety was larger for a worry (vs. positive) episode followed by a positive episode. These results are consistent with the assumptions of the CAM that the function of worry is to avoid negative contrast in emotions that might be triggered by positive experiences or events, which might bring an increase in negative emotions (Newman and Llera, 2011). At the same time, our findings support the notion that worry also has the role to increase the probability of positive contrasts that might follow positive experiences or events, which might, in the end, bring a reduction in negative emotions (Llera and Newman, 2014).

We should note that in the current study, we only measure negative emotions within reported episodes (i.e., anxiety level), and previous research has shown that individuals can simultaneously experience both anxiety and positive emotions or wellbeing (Huppert and Whittington, 2003; Weich et al., 2011). Thus, it could be that the experience of a previous positive episode facilitates more positive emotions in a next worry episode than a previous worry episode might do, without, however, impacting the level of negative emotions in that current worry episode. Future research should integrate an assessment of positive emotions during these episodes and replicate the current findings in GAD and other samples, together with comparing the reliability of different assessment methods across samples. Moreover, although previous episode type and severity did not predict severity in the current episode, it could be that they predict current episode duration. This was beyond the aim of the current research and should be investigated by future studies. Finally, an alternative explanation for the current results is that, although participants were instructed to report worry episodes, the assessed worry episodes could have included rumination or angry thoughts, potentially explaining the lack of significant relationships with anxiety. This might be the case given the fact that various types of repetitive negative thoughts, such as worry and rumination many similar features, e.g., repetitiveness and abstract self-referential mental health activity (Watkins, 2008), and activate common neural networks (Steinfurth et al., 2017). Future research should replicate these preliminary findings assessing other components of emotion (i.e., positive emotions), wellbeing, and emotion regulation in GAD. Moreover, future research should compare the current findings on individuals with GAD to other anxiety disorders in order to better understand common as well as specific manifestations of different anxiety disorders (e.g., Craske et al., 2009; Cisler and Olatunji, 2012).

Their preliminary status acknowledged, the present results do have important clinical implications. Given the emotional reactions to worry and positive episodes in individuals with GAD observed in this study compared to retrospective self-reports that are usually characterized by recall bias (Trull and Ebner-Priemer, 2013), the use of ecological self-monitoring with the aim of collecting in-the-moment information about intense worry episodes and positive experiences, as well as reactions to those in individuals with GAD rather than solely relaying on global retrospective self-reports, is recommend. Indeed, various diary methods are common practice in psychological interventions (Iida et al., 2012). Accurate information about anxiety and controllability levels is essential for establishing a baseline from which to plan interventions and monitor therapeutic change (Mathersul and Ruscio, 2020). Second, momentary measures could also have intervention potential. Monitoring positive experiences and intense worry episodes and ways to deal with those experiences in real time could help individuals with GAD realize that their anxiety and perceived controllability over their experiences are not stable but they are rather fluctuating depending on life contexts and events. Moreover, they can identify activities and behaviors that are associated with lower levels of anxiety and maybe a higher sense of controllability over their experiences and emotions.

The present study has several limitations. First, although memory biases were minimized by sampling emotional reactions to GAD individuals’ worry and positive episodes in real time, the reactions (i.e., anxiety level and perceived controllability of an episode) were still self-reported by participants and may have been colored by negativity biases. Future EMA studies investigating worry and positive experiences in individuals with GAD would benefit from supplementing subjective emotional ratings with more objective measures, such as ambulatory psychophysiology measures (e.g., Schwerdtfeger and Dick, 2019). Second, we assessed self-reported affect as a one-dimensional construct in worry and positive episodes (i.e., the only affect we assessed was anxiety). Therefore, future studies might want to include an assessment of positive affect because high negative affect, such as high anxiety levels, does not automatically imply low positive affect (e.g., Huppert and Whittington, 2003). Third, although we assessed worry episodes, we did not assess worry level corresponding to these episodes.

Fourth, all study prompts were only event contingent (participant-initiated). Participants may not have reported all worry or positive episodes. For example, a participant may have had a worry episode in between a positive and a worry episode but have not reported it. Because of this, we do not know what the anxiety levels were between reported episodes. However, more research is needed to better understand the influence of sampling method (i.e., event-based vs. time-based assessment) on the actual experience. Fifth, although we controlled for time between entries in our analyses, it could be that the non-significant time-lagged effects we found for anxiety are because the time intervals between participants’ self-initiated reports were too long in duration (i.e., the delays could have been too long, and effect of prior episodes weakened). For example, it is possible many participants did not choose to enter rapidly sequenced episodes back-to-back (or did not perceive them as separate, etc.). Participants could also have “worried” briefly or less intensely after a positive episode, yet prior to what they considered to be worthy of being considered a “worry episode”. Therefore, despite statistical control, timing is still a possible confounding variable due to the event-contingent EMA design. Sixth, the GAD sample in the current study was predominantly a female sample. Finally, we did not include a healthy non-GAD comparison group and therefore, it is unclear if the experiences found here are specific to GAD only or true for a wider population.

Limitations notwithstanding, the present study used smartphone-based EMA with an event-based (participant-initiated) approach to examine reactions to worry and positive episodes in the daily lives of individuals with GAD. The focus on assessment of positive experiences is an important and relatively novel contribution to the GAD literature. We found that perceptions of controllability are variable in individuals with GAD; controllability is often not conceptualized this way, seen as more trait-like than state-like. Also, the finding that greater controllability is experienced in positive episodes than worry episodes in GAD has important clinical relevance. Lastly, the findings that worry predicts greater perceptions of controllability in later worry episodes is a novel finding that is not explicitly discussed in the current worry models; it suggests one function of worry may be to confer a sense of controllability to the worrier.

## Data Availability Statement

The raw data supporting the conclusions of this article will be made available by the authors, without undue reservation.

## Ethics Statement

The studies involving human participants were reviewed and approved by the Ethical Committee of University of Zurich (BASEC 2016-00773). The patients/participants provided their written informed consent to participate in this study.

## Author’s Note

Portions of this research were previously presented at the International Meeting of the Society for Psychotherapy Research (June, 2018) and the World Congress of Behavioural and Cognitive Therapies (July, 2019).

## Author Contributions

AV and CF contributed to the conception and design of the study. AV organized the database, performed the statistical analysis, and wrote the first draft of the manuscript. All authors contributed to manuscript revision, read, and approved the submitted version.

## Funding

This study was supported by the Swiss National Science Foundation (grant PP00P1_163702 and PP00P1_190083, recipient: Christoph Flückiger).

## Conflict of Interest

The authors declare that the research was conducted in the absence of any commercial or financial relationships that could be construed as a potential conflict of interest.

## Publisher’s Note

All claims expressed in this article are solely those of the authors and do not necessarily represent those of their affiliated organizations, or those of the publisher, the editors and the reviewers. Any product that may be evaluated in this article, or claim that may be made by its manufacturer, is not guaranteed or endorsed by the publisher.

## Footnotes

^1^ https://xs.movisens.com

^2^Because in the current study, EMA was implemented as an episode-based assessment, namely, the participants were instructed to register an episode, each time one takes place, time between registered episodes was not equal. Therefore, the four models were rerun with time between episodes included in each model. We calculated time between episodes as the time between the end of the previous episode (*t*_0−1_) and the beginning of the current episode (*t*_0_; in minutes). Having the time between episodes included in these models did not change the results, with the exception of Model 4 (see the Results section and Table 2 for more details).

^3^Previous studies investigating worry episodes/frequency in individuals with GAD and found similar worry frequency per day (e.g., Verkuil et al., 2007: *M* = 3.55, *SD* = 3.81, range: 0–30).

^4^In the study of Verkuil et al. (2007), participants worried, on average, during 4.69 days (*SD* = 1.62) when instructed to worry for six consecutive days.

^5^Similar worry duration per day was reported in the study of Verkuil et al. (2007): *M* = 28.04 (*SD* = 41.62, range: 0–524.33).

^6^Additionally, we exploratively tested an interaction effect between previous episode type and previous anxiety/controllability in predicting anxiety and controllability in a current episode. An interaction effect previous controllability level by previous episode type was found only for the model in which controllability in a current positive episode was predicted (for detailed results, see Supplementary Table S1; for a graphical depiction of this interaction effect, see Supplementary Figure S1).

## Supplementary Material

The Supplementary Material for this article can be found at: https://www.frontiersin.org/articles/10.3389/fpsyg.2021.722881/full#supplementary-material

## References

[ref1] Abel J. L. Borkovec T. D. (1995). Generalizability of DSM-III-R generalized anxiety disorders to proposed DSM-IV criteria and cross-validation of proposed changes. J. Anxiety Disord. 9, 303–315. doi: 10.1016/0887-6185(95)00011-C

[ref2] American Psychiatric Association (2013). Diagnostic and Statistical Manual of Mental Disorders. 5th Edn. Washington, DC: American Psychiatric Publishing

[ref3] Backs R. W. da Silva S. P. Han K. (2005). A comparison of younger and older adults' self-assessment manikin ratings of affective pictures. Exp. Aging Res. 31, 421–440. doi: 10.1080/03610730500206808, PMID: 16147461

[ref4] Bates D. Maechler M. Bolker B. Walker S. (2015). Fitting linear mixed-effects models using lme4. J. Stat. Software 67, 1–48. doi: 10.18637/jss.v067.i01

[ref6] Bliese P. (2016). multilevel: Multilevel Functions. R package Version 2.6. Available at: https://CRAN.R-project.org/package=multilevel (Accessed August 23, 2021).

[ref7] Bolger N. Davis A. Rafaeli E. (2003). Diary methods: capturing life as it is lived. Annu. Rev. Psychol. 54, 579–616. doi: 10.1146/annurev.psych.54.101601.145030, PMID: 12499517

[ref8] Bolger N. Laurenceau J. (2013). Intensive Longitudinal Methods: An Introduction to Diary and Experience Sampling Research. New York, NY: Guilford Press.

[ref9] Borkovec T. D. (1994). “The nature, functions, and origins of worry,” in Worrying: Perspectives on Theory, Assessment and Treatment. eds. Davey G. C. L. Tallis F. (Oxford, England: John Wiley & Sons), 5–33.

[ref10] Bottesi G. Ghisi M. Carraro E. Barclay N. Payne R. Freeston M. H. (2016). Revising the intolerance of uncertainty model of generalized anxiety disorder: evidence from UK and Italian undergraduate samples. Front. Psychol. 7:1723. doi: 10.3389/fpsyg.2016.01723, PMID: 27847496 PMC5088195

[ref11] Bradley M. M. Lang P. J. (1994). Measuring emotion: the self-assessment manikin and the semantic differential. J. Behav. Ther. Exp. Psychiatry 25, 49–59. doi: 10.1016/0005-7916(94)90063-9, PMID: 7962581

[ref12] Bylsma L. M. Morris B. H. Rottenberg J. (2008). A meta-analysis of emotional reactivity in major depressive disorder. Clin. Psychol. Rev. 28, 676–691. doi: 10.1016/j.cpr.2007.10.001, PMID: 18006196

[ref13] Bylsma L. M. Taylor-Clift A. Rottenberg J. (2011). Emotional reactivity to daily events in major and minor depression. J. Abnorm. Psychol. 120, 155–167. doi: 10.1037/a0021662, PMID: 21319928

[ref14] Cheavens J. S. Feldman D. B. Woodward J. T. Snyder C. R. (2006). Hope in cognitive psychotherapies: On working with client strengths. J. Cogn. Psychother. 20, 135–145. doi: 10.1891/jcop.20.2.135

[ref15] Cisler J. M. Olatunji B. O. (2012). Emotion regulation and anxiety disorders. Curr. Psychiatr. Rep. 14, 182–187. doi: 10.1007/s11920-012-0262-2, PMID: 22392595 PMC3596813

[ref16] Craske M. G. Rapee R. M. Jackel L. Barlow D. H. (1989). Qualitative dimensions of worry in DSM-III-R generalized anxiety disorder subjects and nonanxious controls. Behav. Res. Ther. 27, 397–402. doi: 10.1016/0005-7967(89)90010-7, PMID: 2775149

[ref17] Craske M. G. Rauch S. L. Ursano R. Prenoveau J. Pine D. S. Zinbarg R. E. (2009). What is an anxiety disorder? Depress. Anxiety 26, 1066–1085. doi: 10.1002/da.20633, PMID: 19957279

[ref18] Crouch T. A. Lewis J. A. Erickson T. M. Newman M. G. (2017). Prospective investigation of the contrast avoidance model of generalized anxiety and worry. Behav. Ther. 48, 544–556. doi: 10.1016/j.beth.2016.10.001, PMID: 28577589

[ref19] Dichter G. S. (2010). Anhedonia in unipolar major depressive disorder: A review. Open Psychiatr. J. 4, 1–9. doi: 10.2174/1874354401004010001

[ref20] Dickson K. S. Ciesla J. A. Reilly L. C. (2012). Rumination, worry, cognitive avoidance, and behavioral avoidance: examination of temporal effects. Behav. Ther. 43, 629–640. doi: 10.1016/j.beth.2011.11.002, PMID: 22697450

[ref21] Duckworth A. Steen T. A. Seligman M. E. (2005). Positive psychology in clinical practice. Annu. Rev. Clin. Psychol. 1, 629–651. doi: 10.1146/annurev.clinpsy.1.102803.144154, PMID: 17716102

[ref22] Dugas M. J. Gagnon F. Ladouceur R. Freeston M. H. (1998). Generalized anxiety disorder: A preliminary test of a conceptual model. Behav. Res. Ther. 36, 215–226. doi: 10.1016/S0005-7967(97)00070-3, PMID: 9613027

[ref23] Ehring T. Watkins E. R. (2008). Repetitive negative thinking as a transdiagnostic process. Int. J. Cogn. Ther. 1, 192–205. doi: 10.1521/ijct.2008.1.3.192

[ref24] Erdfelder E. Faul F. Buchner A. (1996). GPOWER: A general power analysis program. Behav. Res. Methods Instrum. Comput. 28, 1–11. doi: 10.3758/BF03203630

[ref26] Fisher A. J. Bosley H. G. (2020). Identifying the presence and timing of discrete mood states prior to therapy. Behav. Res. Ther. 128:103596. doi: 10.1016/j.brat.2020.103596, PMID: 32135317

[ref27] Flückiger C. Vîslă A. Hilpert P. Wolfer C. Zinbarg R. E. Lutz W.. (2021). Exploring change in cognitive-behavioral therapy for generalized anxiety disorder – A two-arms, patient blinded, ABAB crossed-therapist randomized clinical implementation trial. J. Consult. Clin. Psychol. 89, 454–468. doi: 10.1037/ccp0000639, PMID: 33829819

[ref28] Flückiger C. Wüsten G. Zinbarg R. E. Wampold B. E. (2010). Resource Activation – Using Clients’ Own Strengths in Psychotherapy and Counseling. Cambridge, MA: Hogrefe.

[ref29] Flückiger C. Zinbarg R. E. Znoj H. J. Ackert M. (2014). Resource activation in generalized anxiety disorder: An observer-based microprocess analysis of in-session outcomes. Psychotherapy 51, 535–545. doi: 10.1037/a0034119, PMID: 24341894

[ref30] Fredrickson B. L. (2001). The role of positive emotions in positive psychology: The broaden-and-build theory of positive emotions. Am. Psychol. 56, 218–226. doi: 10.1037/0003-066X.56.3.218 11315248 PMC3122271

[ref31] Gelso C. J. Woodhouse S. (2003). “Toward a positive psychotherapy: focus on human strength,” in Counseling Psychology and Optimal Human Functioning. ed. Walsh W. B. (Malwah, NJ: Lawrence Erlbaum), 171–196.

[ref32] Grawe K. (2006). Neuropsychotherapy. New York: Taylor & Francis.

[ref33] Hallion L. S. Ruscio A. M. (2013). Should uncontrollable worry be removed from the definition of GAD? A test of incremental validity. J. Abnorm. Psychol. 122, 369–375. doi: 10.1037/a0031731, PMID: 23713499

[ref34] Hamaker E. L. (2012). “Why researchers should think “within-person” a paradigmatic rationale,” in Handbook of Research Methods for Studying Daily Life. eds. Mehl M. R. Conner T. S. (New York, NY: Guilford Publications), 43–61.

[ref35] Higgins E. T. (1998). “Promotion and prevention: regulatory focus as a motivational principle,” in Advances in Experimental Social Psychology. ed. Zanna M. P., Vol. 30 (New York: Academic Press), 1–46.

[ref36] Hirsch C. R. Mathews A. Lequertier B. Perman G. Hayes S. (2013). Characteristics of worry in generalized anxiety disorder. J. Behav. Ther. Exp. Psychiatry 44, 388–395. doi: 10.1016/j.jbtep.2013.03.004, PMID: 23651607 PMC3743042

[ref37] Hoyer J. Becker E. S. Roth W. T. (2001). Characteristics of worry in GAD patients, social phobics, and controls. Depress. Anxiety 13, 89–96. doi: 10.1002/da.1021, PMID: 11301925

[ref38] Huppert F. A. Whittington J. E. (2003). Evidence for the independence of positive and negative well-being: implications for quality of life assessment. Br. J. Health Psychol. 8, 107–122. doi: 10.1348/135910703762879246, PMID: 12643820

[ref39] Hur J. Gaul K. Berenbaum H. (2019). Different patterns of attention bias in worry and rumination. Cogn. Ther. Res. 43, 713–725. doi: 10.1007/s10608-018-09993-4

[ref40] Iida M. Shrout P. E. Laurenceau J.-P. Bolger N. (2012). “Using diary methods in psychological research,” in APA Handbooks in Psychology^®^. APA Handbook of Research Methods in Psychology, Vol. 1. Foundations, Planning, Measures, and Psychometrics. eds. Cooper H. Camic P. M. Long D. L. Panter A. T. Rindskopf D. Sher K. J. (Washington, DC: American Psychological Association), 277–305.

[ref41] Khazanov G. K. Ruscio A. M. Swendsen J. (2019). The “brightening” effect: reactions to positive events in the daily lives of individuals with major depressive disorder and generalized anxiety disorder. Behav. Ther. 50, 270–284. doi: 10.1016/j.beth.2018.05.008, PMID: 30824245 PMC6494459

[ref42] LaFreniere L. S. Newman M. G. (2019). The impact of uncontrollability beliefs and thought-related distress on ecological momentary interventions for generalized anxiety disorder: A moderated mediation model. J. Anxiety Disord. 66:102113. doi: 10.1016/j.janxdis.2019.102113, PMID: 31362145 PMC6692212

[ref43] Llera S. J. Newman M. G. (2010). Effects of worry on physiological and subjective reactivity to emotional stimuli in generalized anxiety disorder and nonanxious control participants. Emotion 10, 640–650. doi: 10.1037/a0019351, PMID: 21038947

[ref44] Llera S. J. Newman M. G. (2014). Rethinking the role of worry in generalized anxiety disorder: evidence supporting a model of emotional contrast avoidance. Behav. Ther. 45, 283–299. doi: 10.1016/j.beth.2013.12.011, PMID: 24680226

[ref01] Margraf J. Cwik J. C. Suppiger A. Schneider S. (2017). DIPS Open Access: Diagnostic Intervew for Mental Disorders [DIPS Open Access: Diagnostisches Interview bei psychischen Störungen]. Ruhr-Universität Bochum. Available at: http://dips-interviews.rub.de

[ref45] Mathersul D. C. Ruscio A. M. (2020). Forecasting the future, remembering the past: misrepresentations of daily emotional experience in generalized anxiety disorder and major depressive disorder. Cogn. Ther. Res. 44, 73–88. doi: 10.1007/s10608-019-10048-5

[ref46] McEvoy P. M. Erceg-Hurn D. M. Anderson R. A. Campbell B. N. Nathan P. R. (2015). Mechanisms of change during group metacognitive therapy for repetitive negative thinking in primary and non-primary generalized anxiety disorder. J. Anxiety Disord. 35, 19–26. doi: 10.1016/j.janxdis.2015.07.003, PMID: 26311192

[ref02] McLaughlin K. A. Borkovec T. D. Sibrava N. J. (2007). TThe effects of worry and rumination on affect states and cognitive activity. Behav. Ther. 38, 23–38. doi: 10.1016/j.beth.2006.03.003 17292692

[ref47] Mineka S. Zinbarg R. (2006). A contemporary learning theory perspective on the etiology of anxiety disorders: It's not what you thought it was. Am. Psychol. 61, 10–26. doi: 10.1037/0003-066X.61.1.10 16435973

[ref48] Moore R. C. Depp C. A. Wetherell J. L. Lenze E. J. (2016). Ecological momentary assessment versus standard assessment instruments for measuring mindfulness, depressed mood, and anxiety among older adults. J. Psychiatr. Res. 75, 116–123. doi: 10.1016/j.jpsychires.2016.01.011, PMID: 26851494 PMC4769895

[ref49] Newman M. G. Jacobson N. C. Zainal N. H. Shin K. E. Szkodny L. E. Sliwinski M. J. (2019). The effects of worry in daily life: An ecological momentary assessment study supporting the tenets of the contrast avoidance model. Clin. Psychol. Sci. 7, 794–810. doi: 10.1177/2167702619827019, PMID: 31372313 PMC6675025

[ref50] Newman M. G. Llera S. J. (2011). A novel theory of experiential avoidance in generalized anxiety disorder: A review and synthesis of research supporting a contrast avoidance model of worry. Clin. Psychol. Rev. 31, 371–382. doi: 10.1016/j.cpr.2011.01.008, PMID: 21334285 PMC3073849

[ref51] Newman M. G. Przeworski A. (2018). The increase in interest in GAD: commentary on Asmundson & Asmundson. J. Anxiety Disord. 56, 11–13. doi: 10.1016/j.janxdis.2018.04.006, PMID: 29859657 PMC7340183

[ref55] Peeters F. Nicolson N. A. Berkhof J. Delespaul P. deVries M. (2003). Effects of daily events on mood states in major depressive disorder. J. Abnorm. Psychol. 112, 203–211. doi: 10.1037/0021-843X.112.2.203, PMID: 12784829

[ref53] Rapee R. M. Craske M. G. Brown T. A. Barlow D. H. (1996). Measurement of perceived control over anxiety-related events. Behav. Ther. 27, 279–293. doi: 10.1016/S0005-7894(96)80018-9

[ref54] Rashid T. Ostermann R. F. (2009). Strength-based assessment in clinical practice. J. Clin. Psychol. 65, 488–498. doi: 10.1002/jclp.20595, PMID: 19294732

[ref56] Ruscio A. M. Borkovec T. D. (2004). Experience and appraisal of worry among high worriers with and without generalized anxiety disorder. Behav. Res. Ther. 42, 1469–1482. doi: 10.1016/j.brat.2003.10.007, PMID: 15500816

[ref57] Scheel M. J. Davis C. K. Henderson J. D. (2013). Therapist use of client strengths: A qualitative study of positive processes. Couns. Psychol. 41, 392–427. doi: 10.1177/0011000012439427

[ref58] Schwerdtfeger A. R. Dick K. (2019). Episodes of momentary resilience in daily life are associated with HRV reductions to stressful operations in firefighters: an ambulatory assessment approach using bayesian multilevel modeling. J. Posit. Psychol. 14, 593–602. doi: 10.1080/17439760.2018.1497689

[ref59] Seligman M. E. P. Peterson C. (2003). “Positive clinical psychology,” in A Psychology of Human Strengths: Fundamental Questions and Future Directions for a Positive Psychology. eds. Aspinwall L. G. Staudinger U. M. (Washington, DC: American Psychological Association), 305–317.

[ref60] Shiffman S. Stone A. A. Hufford M. R. (2008). Ecological momentary assessment. Annu. Rev. Clin. Psychol. 4, 1–32. doi: 10.1146/annurev.clinpsy.3.022806.091415, PMID: 18509902

[ref61] Solhan M. B. Trull T. J. Jahng S. Wood P. K. (2009). Clinical assessment of affective instability: comparing EMA indices, questionnaire reports, and retrospective recall. Psychol. Assess. 21, 425–436. doi: 10.1037/a0016869, PMID: 19719353 PMC2864015

[ref62] Steinfurth E. C. Alius M. G. Wendt J. Hamm A. O. (2017). Physiological and neural correlates of worry and rumination: support for the contrast avoidance model of worry. Psychophysiology 54, 161–171. doi: 10.1111/psyp.12767, PMID: 27766641

[ref63] Strauman T. J. Socolar Y. Kwapil L. Cornwell J. F. Franks B. Sehnert S.. (2015). Microinterventions targeting regulatory focus and regulatory fit selectively reduce dysphoric and anxious mood. Behav. Res. Ther. 72, 18–29. doi: 10.1016/j.brat.2015.06.003, PMID: 26163353 PMC4529777

[ref64] Thielsch C. Andor T. Ehring T. (2015). Do metacognitions and intolerance of uncertainty predict worry in everyday life? An ecological momentary assessment study. Behav. Ther. 46, 532–543. doi: 10.1016/j.beth.2015.05.001, PMID: 26163716

[ref65] Thompson R. J. Mata J. Jaeggi S. M. Buschkuehl M. Jonides J. Gotlib I. H. (2012). The everyday emotional experience of adults with major depressive disorder: examining emotional instability, inertia, and reactivity. J. Abnorm. Psychol. 121, 819–829. doi: 10.1037/a0027978, PMID: 22708886 PMC3624976

[ref66] Trull T. J. Ebner-Priemer U. (2013). Ambulatory assessment. Annu. Rev. Clin. Psychol. 9, 151–176. doi: 10.1146/annurev-clinpsy-050212-185510 23157450 PMC4249763

[ref68] Verkuil B. Brosschot J. F. Thayer J. F. (2007). Capturing worry in daily life: are trait questionnaires sufficient? Behav. Res. Ther. 45, 1835–1844. doi: 10.1016/j.brat.2007.02.004, PMID: 17382896

[ref69] Wahl K. Ehring T. Kley H. Lieb R. Meyer A. Kordon A.. (2019). Is repetitive negative thinking a transdiagnostic process? A comparison of key processes of RNT in depression, generalized anxiety disorder, obsessive-compulsive disorder, and community controls. J. Behav. Ther. Exp. Psychiatry 64, 45–53. doi: 10.1016/j.jbtep.2019.02.006, PMID: 30851652

[ref70] Watkins E. R. (2008). Constructive and unconstructive repetitive thought. Psychol. Bull. 134, 163–206. doi: 10.1037/0033-2909.134.2.163, PMID: 18298268 PMC2672052

[ref71] Wells A. Carter K. (2001). Further tests of a cognitive model of generalized anxiety disorder: metacognitions and worry in GAD, panic disorder, social phobia, depression, and nonpatients. Behav. Ther. 32, 85–102. doi: 10.1016/S0005-7894(01)80045-9

[ref72] Wetherell J. L. Le Roux H. Gatz M. (2003). DSM-IV criteria for generalized anxiety disorder in older adults: distinguishing the worried from the well. Psychol. Aging 18, 622–627. doi: 10.1037/0882-7974.18.3.622, PMID: 14518822

[ref73] Weich S. Brugha T. King M. McManus S. Bebbington P. Jenkins R.. (2011). Mental well-being and mental illness: findings from the adult psychiatric morbidity survey for England 2007. Br. J. Psychiatry 199, 23–28. doi: 10.1192/bjp.bp.111.091496, PMID: 21719878

[ref74] Wickham H. François R. Henry L. Müller K. (2019). dplyr: A grammar of data manipulation. R package Version 0.8.3. Available at: https://CRAN.R-project.org/package=dplyr (Accessed August 23, 2021).

