# Supplementary material for: Worry and Positive Episodes in the Daily Lives of Individuals With Generalized Anxiety Disorder: An Ecological Momentary Assessment Study
Source: Front Psychol. 2021 Oct 5;12:722881. doi: 10.3389/fpsyg.2021.722881 (PMC8579489; doi:10.3389/fpsyg.2021.722881)
Supplement: Supplementary file 1 [file Table_1.DOCX]

Table S1.

*Multilevel models*’ *results for the interaction between previous episode type and previous anxiety/controllability in predicting anxiety and controllability in the current episode.*

|  | *γ* | *SE* | *t* | *p* |
| --- | --- | --- | --- | --- |
|  |  |  |  |  |
| Anxiety in worry episodes t_0_ (H2a)^a^ |  |  |  |  |
| Intercept | 58.84 | 4.19 |  |  |
| Anxiety t_0-1_ | 0.16 | 0.11 | 1.46 | 0.147 |
| Episode t_0-1_ | -4.30 | 5.24 | -0.82 | 0.412 |
| Anxiety t_0-1_*Episode t_0-1_ | 0.01 | 0.12 | 0.05 | 0.964 |
| Controllability in worry episodes t_0_ (H2b) ^b^ |  |  |  |  |
| Intercept | 2.53 | 0.74 |  |  |
| Controllability t_0-1_ |  | 0.12 | 1.65 | 0.101 |
| Episode t_0-1_ | -0.12 | 0.78 | -0.16 | 0.872 |
| Controllability t_0-1_*Episode t_0-1_ | 0.10 | 0.13 | 0.78 | 0.436 |
| Anxiety in positive episodes t_0_ (H2c)^c^ |  |  |  |  |
| Intercept | 24.31 | 4.21 |  |  |
| Anxiety t_0-1_ | 0.19 | 0.11 | 1.66 | 0.090 |
| Episode t_0-1_ | 0.83 | 5.47 | 0.15 | 0.080 |
| Anxiety t_0-1_*Episode t_0-1_ | -0.21 | 0.13 | -1.64 | 0.104 |
| Controllability in positive episodes t_0_ (H2d)^d^ |  |  |  |  |
| Intercept | 2.56 | 0.82 |  |  |
| Controllability t_0-1_ | 0.61 | 0.12 | 5.07 | <0.001 |
| Episode t_0-1_ | 4.02 | 0.87 | 4.64 | <0.001 |
| Controllability t_0-1_*Episode t_0-1_^e^ | -0.59 | 0.14 | -4.09 | <0.001 |

*Note.* *Note.* t_0_ = current episode; t_-1_ = previous episode; Episode t_-1_ = type of the previous episode (i.e., positive episode = 0; worry episode = 1).

^a^ H1: Prediction of anxiety experienced in a current worry episode (303 observations from 44 individuals).

^b^ H2: Prediction of controllability experienced in a current worry episode (303 observations from 44 individuals).

^c^ H3: Prediction of anxiety experienced in a current positive episode (162 observations from 39 individuals).

^d^ H4: Prediction of controllability experienced in a current positive episode (162 observations from 39 individuals).

^e^ Having the time between previous and current episode included in these models did not change the results of the four models.
