# Supplementary material for: Worry and Positive Episodes in the Daily Lives of Individuals With Generalized Anxiety Disorder: An Ecological Momentary Assessment Study
Source: Front Psychol. 2021 Oct 5;12:722881. doi: 10.3389/fpsyg.2021.722881 (PMC8579489; doi:10.3389/fpsyg.2021.722881)
Supplement: Supplementary file 2 [file Image_1.pdf]

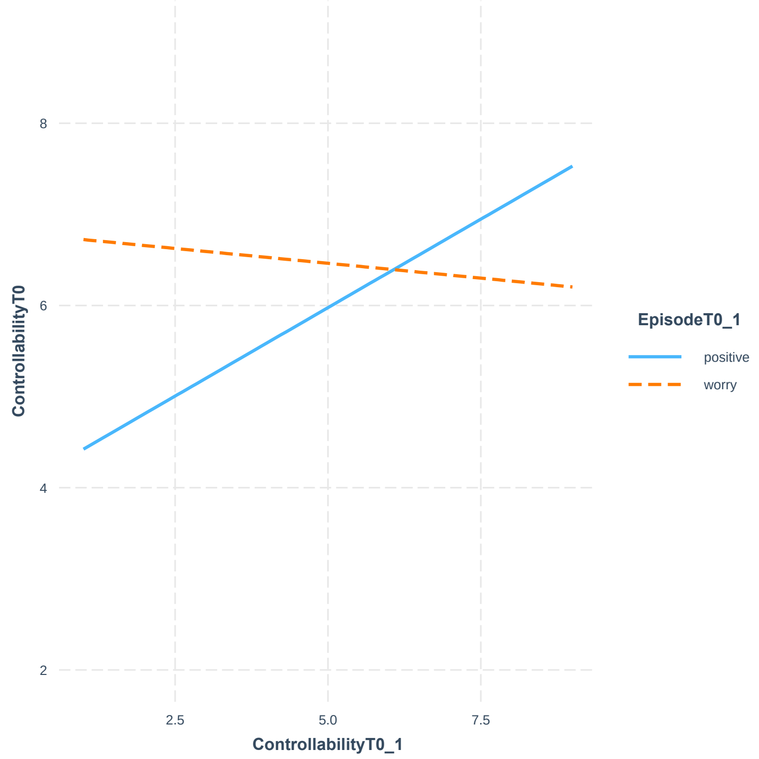

Figure S1. Prediction of controllability in a current positive episode (H2d). ControllabilityT0 = controllability in a current positive episode; ControllabilityT0_1 = controllability in a previous episode.
